# Differential perceptions of teamwork, focused work and perceived productivity as an effect of desk characteristics within a workplace layout

**DOI:** 10.1371/journal.pone.0250058

**Published:** 2021-04-28

**Authors:** Kerstin Sailer, Petros Koutsolampros, Rosica Pachilova

**Affiliations:** The Bartlett School of Architecture, University College London, London, United Kingdom; Peking University Shenzhen Graduate School, CHINA

## Abstract

The impact of the physical workplace on behaviors and attitudes at work is a much-studied topic. Major research streams over the last decades investigated either satisfaction with offices in relation to physical comfort, or how layout decisions influenced interaction and collaboration in the workplace with a focus on open-plan offices. Rather little is known on the effect a workplace layout (such as its openness) has on perceptions of staff regarding teamwork, focused work and perceived productivity. We aim to close this gap by taking a differential approach which appreciates detailed variations within open-plan offices. Not every corner of an office is the same, so the question arises whether satisfaction with workspace differs depending on where someone is sitting. Bringing results of a staff survey in the UK headquarters of a global technology company together with a detailed analysis of spatial qualities at desks based on isovist and visual field analysis, we find that staff are less likely to rate their workplace environment favorably when they have higher numbers of desks within their own field of vision; and when they are facing away from the room with a relatively larger area behind their back compared to the area surrounding them. Aspects of teamwork that are negatively affected include sharing information with others, as well as team identity and cohesion. Focused work (concentration) and working productively are impacted even more so with the largest effect sizes throughout. These findings highlight the relevance of investigating detailed spatial qualities of micro-locations in workplace layouts. Our results also raise important questions regarding the current popular practice in workplace design of providing large open-plan offices for technology companies.

## Introduction

Satisfaction at work is a much-studied topic. Going back to early human factors studies in the 1950s and 1960s as a foundation [1, 2], there is a rich research tradition of assessing worker’s perceptions of and attitudes towards work. Most of those studies considered job satisfaction [3, 4] and gave no thought to the physical space of the office. Studies that included the physical reality of office buildings in relation to job satisfaction overwhelmingly focused on environmental factors such as thermal and acoustic comfort, lighting or indoor air quality [5–7].

Yet, another aspect of the physical workplace has been hotly debated by scholars over the last decades in relation to workplace satisfaction, and that is the question of office layout. The continued discussion on the advantages and disadvantages of open-plan versus cellular office accommodation has not reached consensus, however, with inconsistencies and contradictions as the norm [8, 9]. For instance, some research found that satisfaction with communication did not suffer after a move of one organization from a partitioned environment into open-plan [10]. In contrast, however, more often job satisfaction correlated with privacy perceptions and the degree of office partitioning across a variety of settings [11–13]. A large-scale study concluded that satisfaction was severely compromised in open-plan offices [14].

Other studies investigated the related concern of actual observed increases or decreases of interaction and communication in relation to workplace layout. Here, similar contradictions are apparent as in the discussion on workplace satisfaction.

Evidence was found for both propositions: that partitioned offices were associated with higher degrees of interactions [15], but also that the same was the case for open-plan layouts. This was particularly the cases for high levels of visibility between corridor and workstations since that resulted in an increase in random encounters via the observed mechanism of ‘recruitment’, i.e. the initiation of interactions between people passing by a workspace and those sitting there [16]. Similarly, more integrated areas in floor plans were associated with higher levels of interactions and encounter frequencies [17, 18]. In contrast, more recent studies on open-plan offices again emphasized negative outcomes for collaboration such as reduced face-to-face collaboration [19] and active avoidance of new collaborations [20].

### A differential approach to workplaces

This paper aims to fill a gap in the literature by adopting a differential approach to workplaces. It suggests conceptualizing, analyzing and evaluating detailed workplace layout decisions and their effects on office worker satisfaction by drawing on space syntax as a theory and methodology [21, 22].

This stance is grounded in the proposition that one of the main problems of the ‘scattered empirical evidence’ base [23] as highlighted above is that the discourse conflates differences between various forms, sizes, and shapes of open-plan layouts. To give an example, in their study of more than 40,000 respondents, Kim and de Dear [14] summarized the positive and negative impacts on occupant satisfaction for those in open-plan accommodation, but clustered three different office settings together: cubicles with high partitions, cubicles with low partitions and completely open-plan offices. In addition, only less than 7% of their US-based sample were from open-plan layouts without partitioning. Therefore, well-known issues of cubicles were conflated with settings that arguably varied. Differentiating the degree of partitioning in fact played a major role in early works substantiating the field in the 1980s, since partitioning was hypothesized to matter for worker satisfaction and was classified on a five point scale (according to the numbers of sides enclosed) and used as an independent variable in statistical models [11, 13].

Therefore, it is argued that not every open-plan office is the same. Three different strands of research can be brought to bear: firstly, research considering size of open-plan spaces, as well as desk allocations; secondly, research discussing size and shapes of floor plates; and finally, studies investigating detailed configurations such as quality and type of circulation spaces.

To elaborate on the first strand, extant research showed that job satisfaction ratings and occupant health differed between office workers in the same type of accommodation such as open-plan, when other crucial factors varied [24]. The main factors included in the study were size of the office and desk allocation. Medium-sized open-plan offices (10–24 people in a room) posed higher risks to occupant satisfaction and health than small (4–9 people) and large (>24 people) open-plan offices. Flex offices, defined as those with non-allocated desks, where office workers choose work settings depending on the task at hand consistently showed low risks for health and job satisfaction, even though they were open-plan, too.

The second strand of research differentiated open-plan offices regarding size and shape of floor plates. Building on the seminal work of Thomas Allen, who argued that frequent communication among office workers followed a distance curve and often broke down completely with a change in floor [25], it appears plausible that large floor plates create different affordances than smaller ones. Technology companies in Silicon Valley have identified this effect and implemented mostly horizontal office structures, avoiding multi-level buildings in order to foster collaboration. Most famously among them are the Facebook headquarters in Menlo Park with almost 3,000 employees on a single floor [26] and the Apple headquarters with 12,000 staff across four floors [27]. In addition to size, the shape of the floor plate also appears to matter, as was shown by a study [28] comparing a sample of 50 different office floors according to their morphological properties. The authors argued that the floor plate shapes affected the internal layout of the offices and thus the opportunities of the floor plate to integrate staff to a higher degree. Floor plates that were shaped as compact blocks or bars tended to reach higher integration levels if they implemented a grid-like interior circulation system, whereas more fragmented floor plate shapes, such as pavilion or wing shapes led to lower integration potential. Wing shapes (L, F or U shapes) could be made more integrated by a fishbone like internal circulation system. Integration is meaningful since previous research has highlighted how integrated layouts raise encounter levels among staff [17, 18, 29], however, the relation between integrated layouts and workplace satisfaction is less well established.

Finally, the third strand of research on differentiating office accommodation by detailed configuration provided evidence that the quality of circulation spaces affected office worker behaviors. Corridors with high intervisibility towards workstations led to higher encounter rates among staff by bringing those on the move together with those seated [16]. However, those types of corridors were also shown to provide negative side-effects due to high levels of distractions and disturbances, complaints and low satisfaction for those workers sitting adjacent to the main circulation [9]. This even led to a lower degree of interactions around high use corridors, possibly due to staff actively avoiding further conversations and thus managing distractions.

### Study focus

To summarize, there is a good case to be made to investigate detailed office configurations beyond typological descriptions of a workplace as ‘open-plan’. As was shown above, however, the majority of research on detailed configurations has focused on a whole range of employee outcomes, including frequencies of encounter among staff, collaborative behaviors or health outcomes rather than on satisfaction. The relationship between workplace configurations and job satisfaction continues to be a knowledge gap.

Therefore, this paper chooses a clear focus on staff perception of and satisfaction with an open-plan workplace. It will investigate detailed office configurations on a micro-level, i.e. focusing on the perception of staff while sitting at their desk. In particular, the focus will move away from environmental comfort, which has been addressed by ample research in the past. Instead, the main factors to consider will be perception of teamwork and collaboration with others, as well as perceptions of staff regarding focused work and productivity.

To date only a selective number of research studies has investigated perceptions of teamwork and focused work in relation to detailed layout choices. This is important, since collaboration is a crucial element of knowledge work [30] in the post-industrial society. Arguably both collaboration and concentration are needed for work in the 21^st^ century.

A series of studies on teamwork and focused work perception are worth mentioning in this context. A recent study developed a framework to test employee perceptions to the physical work environment and found that the sense of connectedness of employees as well as their ability to focus predicted collaborations [31]. Connectedness included items such as ‘the workplace allows me to feel part of the organization’ and ‘to see myself as a member of a community’ whereas focus measured whether the workplace allowed staff ‘to concentrate when I need to’ and ‘to control distractions’. It was also shown that the framework measured employee reactions reliably. This means that perceptions of relations to others but also perceptions of focus are important aspects of the work environment.

Similar aspects were investigated by an earlier study of eleven administrative offices in the US [32]. Perceived support from the work environment for collaboration and perceived distraction from interactions were found to vary with layout variations. The study is an interesting contribution, since it distinguishes workstation related measures (partitioning, size of workstation, density, distance to next coworker, presence of a door) from layout-scale measures (distances of workstations to several amenities, percentage of floor space dedicated to amenities). The main factors were identified as distance to amenities as well as degree of floor plan openness (which lowered perceived support for collaboration), while distractions were fueled by closeness to photocopiers. This means that measures derived from the overall layout of the workspace contributed more to the perception of collaboration and focused work than workstation specific ones.

As part of their inquiry into satisfaction and health outcomes, Danielsson and Bodin included one item in their survey which covered teamwork. They found significantly higher odds for lack of good cooperation in medium-sized open-plan offices [24].

All three studies cited above highlight that teamwork perceptions but also perceptions of focused work are relevant for collaboration and seem to vary with office configuration.

Studies using the research framework of space syntax found that satisfaction of office workers with interaction support correlates significantly with spatial integration, i.e. how accessible corridors next to workstations are in the overall spatial system of the floor plan [33]. Yet correlation coefficients were rather low with less than 3% of the variation in perceived interaction support associated with spatial configuration.

Bringing layout-related variables of space syntax back to the level of the individual workstation, a study of judges’ offices explored visibility relations and exposure of staff to coworkers [34]. Due to a small sample size and methodological issues results remained mixed and inconclusive, yet the approach of exploring desk positions and visible areas is noteworthy.

In summary, very few studies explore the relationship between satisfaction with collaborative and focused work on the one hand and workplace configuration on the other, yet those that do find evidence that the layout of workplaces makes a difference to staff perceptions. While early studies focused on workstation relevant measures [10–13], space syntax and configurational research [32, 33] showed that the overall layout played a significant role as well. The focus of this study is to combine space syntax related measurements with the investigation of desk positions of individual workstations.

### Research questions and hypotheses

Finally, this leads to the specific research question that this paper explores, i.e. do office workers perceive teamwork, focused work and productivity differently according to the spatial characteristics of their desk?

To create specific hypotheses, we follow leads from extant literature. Based on the research of Allen [25] it could be expected to find that office workers with a desk in larger areas are more satisfied with connecting with others, since a higher number of coworkers are immediately available. Likewise, higher levels of density mean potentially more people in close proximity. This leads to hypothesis 1a-b:

**H1:** Workers with desks surrounded by higher numbers of other desks **(a)** or in a higher density area **(b)** are more likely to rate their office environment as favorable regarding teamwork (accessing coworkers, sharing information, spontaneous and planned meetings and getting to know people).

Visibility provides a sense of awareness for other people, as argued by Allen and Henn [35]. If workers have eye contact to their coworkers, this heightens their awareness for the co-presence of others, eventually supporting communication and sharing of knowledge, according to the authors. Thus, we hypothesize:

**H2:** Workers with desks surrounded by higher numbers of other desks are more likely to rate their office environment as favorable regarding awareness of their own team and awareness of other teams.

Building on Danielsson and Bodin’s work [24] as well as early work by Sundstrom and Oldham [11–13] it could also be the case that office workers in smaller neighborhoods, i.e. those sharing an open-plan area with fewer people, are more satisfied with the cohesion and identity of their team as well as their ability to concentrate and work productively. Kim and De Dear also highlighted a lack of visual privacy as one of the leading reasons for workplace dissatisfaction [14]. Visual privacy can be argued to relate both directly to the forward-facing viewshed and indirectly to the size of an office neighborhood, as smaller areas provide less exposure to other people passing by. Therefore, we hypothesize:

**H3:** Workers with desks surrounded by lower numbers of other desks **(a)** or a lower number of other desks in their direct, forward-facing viewshed **(b)** are more likely to rate their office environment as favorable regarding team identity and cohesion, concentrating and working productively.

Research also suggests that not only the numbers of potential people in a space might matter, but also one’s location in relation to them. A study of different customer clubs [36] highlighted that seats with a higher degree of a protected back, e.g. against a wall, were preferred due to offering a place to observe others without being exposed, an outcome that agrees with the theory of prospect and refuge [37]. Therefore, it could be expected that people at a desk with a relatively high ratio of view in front of them versus what lies behind them will feel more in control and able to connect with others more easily. It could also be the case that simply the numbers of people in forward-facing view matters rather than the ratio of what is in front and what is behind people. Thus, we hypothesize:

**H4:** Workers facing the room, i.e. with larger visible areas in front of them compared to the size of the whole office area surrounding them **(a)** or a higher number of other desks in their direct, forward-facing viewshed **(b)** are more likely to rate their environment as favorable regarding teamwork (accessing coworkers, sharing information, spontaneous and planned meetings and getting to know people).

Finally, research showing the positive and negative effects of heavily used circulation spaces can be drawn upon. Following Backhouse and Drew [16] it could be expected that people with a seat directly adjacent to the corridor are more satisfied with connecting with others, while following Sailer’s [9] insights would suggest that a seating position close to the corridor has a negative effect on satisfaction with concentration and productive working. Research also highlighted that occupants seated directly next to a window showed higher levels of satisfaction with their workplace [38]. This leads to the following hypotheses:

**H5a:** Workers with desks next to a corridor are more likely to rate their environment as favorable regarding teamwork (accessing coworkers, sharing information, spontaneous and planned meetings and getting to know people).
**H5b:** Workers with desks next to a window are more likely to rate their environment as favorable regarding concentration and working productively.

In summary, this means the following five characteristics of a desk will be studied: 1) how many other desks it is surrounded by; 2) how densely packed the surrounding area is; 3) how many desks are seen in a forward-facing view; 4) the ratio between forward facing viewshed area and overall surroundings; 5) seat type by micro-location of desk, i.e. next to a window, corridor, wall or mid-row. The next section will now explain the methods and all metrics employed in this study in more detail.

## Methods

### Study context

**Fig 1:**
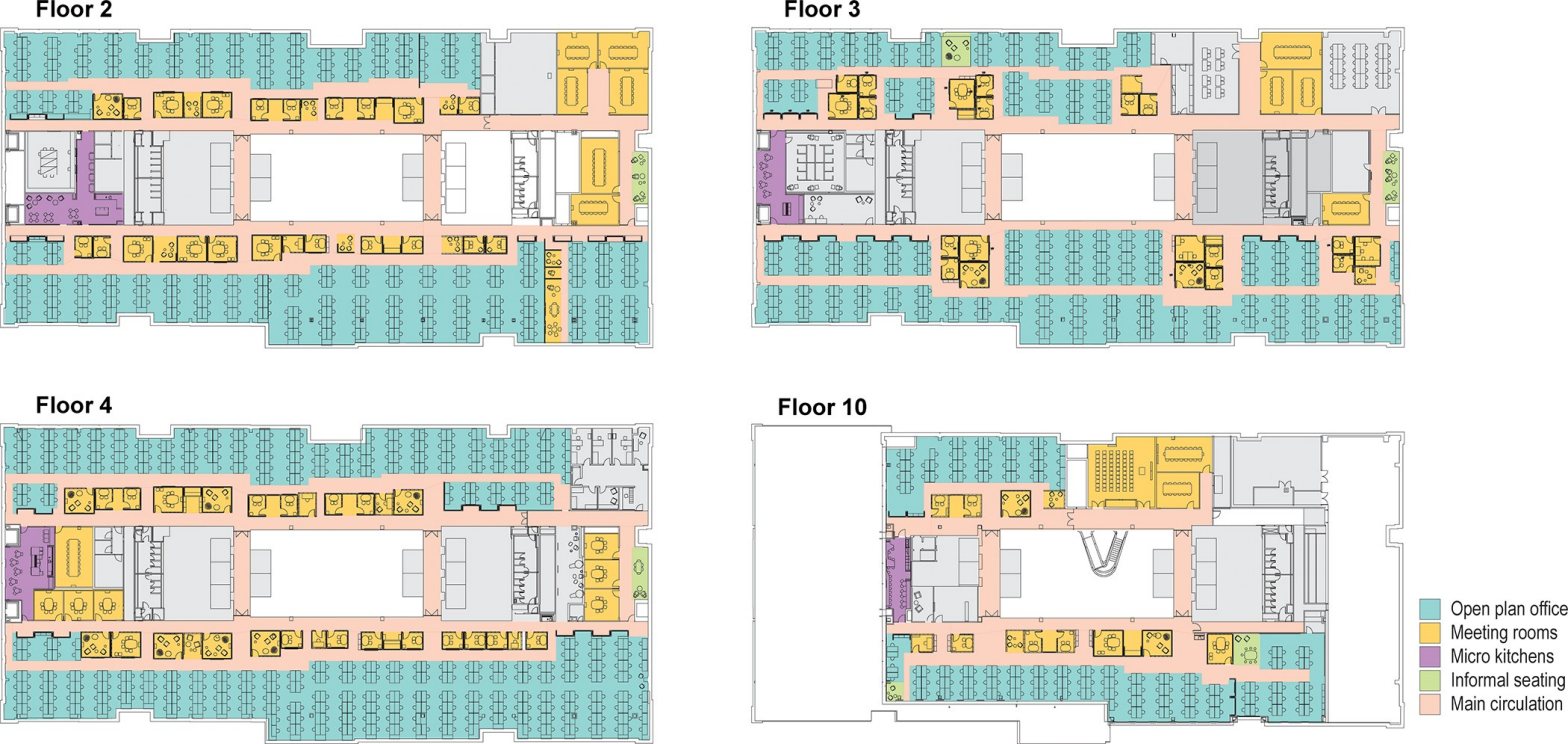
Annotated floor plan of the four floors of office accommodation at ‘Tech’ that were included in the study.

This research was undertaken in the UK headquarters of a large international technology company, called ‘Tech’ in the following. The London-based office housed several thousand staff in a single multilevel building, which had been designed by an architectural firm in 2016. Four office floors were included in the study, as shown in Fig 1.

### Ethics

All human participants gave fully informed written consent before participating in the study. No personal identifiers were collected. Responses were only shared with the company in aggregate form. The study was approved by the UCL Research Ethics Committee (reference number 6118–003).

### Data collection and sample

Fieldwork was undertaken in spring 2018. Two main data sets were collected: 1) responses to a staff questionnaire and 2) office seating positions of participants.

The staff questionnaire was issued online and sent to all staff on the participating floors by administrators of ‘Tech’. It comprised of a series of questions on satisfaction with workspaces and meeting rooms. The main question used in this paper is ‘How much does the workspace at ‘Tech’ support or inhibit the below listed aspects of working life?’ Answers included ‘sharing information with others quickly’, ‘knowing what is going on in your team / other teams’, ‘your team’s identity and cohesion’, ‘concentrating on tasks’, etc. Participants were asked to rate each item on a 7-point Likert scale from extremely supportive (+3), very supportive (+2), fairly supportive (+1), neutral (0) to fairly inhibitive (-1), very inhibitive (-2) and extremely inhibitive (-3). A full overview of items is provided in S1 File. 172 answers were collected, i.e. the response rate was 16%.

We also asked participants for their length of experience in the technology industry, their tenure of working for ‘Tech’ as well as their role at ‘Tech’. Gender and age attributes of workers were not collected in the survey in line with a minimal data capture practice. Team affiliation was initially requested, but not used in further analysis as it was ill-defined with team sizes ranging from 1 to 225, thereby mixing up smaller teams and larger departmental structures.

Seating positions of participants were obtained through the online staff questionnaire by asking staff to provide their seat asset number, which was found on a sticker on their desk. The researchers received a floor plan marked with those numbers. The seat categorization (mid row, next to wall, next to window, next to corridor) was undertaken by the research team. Visible areas from desk positions were analyzed using space syntax techniques. In particular, isovist analysis was used, calculating the visible area from a specific vantage point [39]. More details are provided below in the overview of detailed metrics.

### Statistical analysis

The analysis of isovists, i.e. size of visible areas from each office desk as a vantage point was undertaken using depthmapX [40]. Further data processing and statistical analysis was completed in R [41]. For the analysis in R various packages were used, namely rgeos [42], rgdal [43], maptools [44] and dxfspatial [45] for the spatial data processing and analysis, as well as base R, ggplot2 [46], GGally [47], and png [48] for plotting.

### Overview of spatial metrics

**Fig 2:**
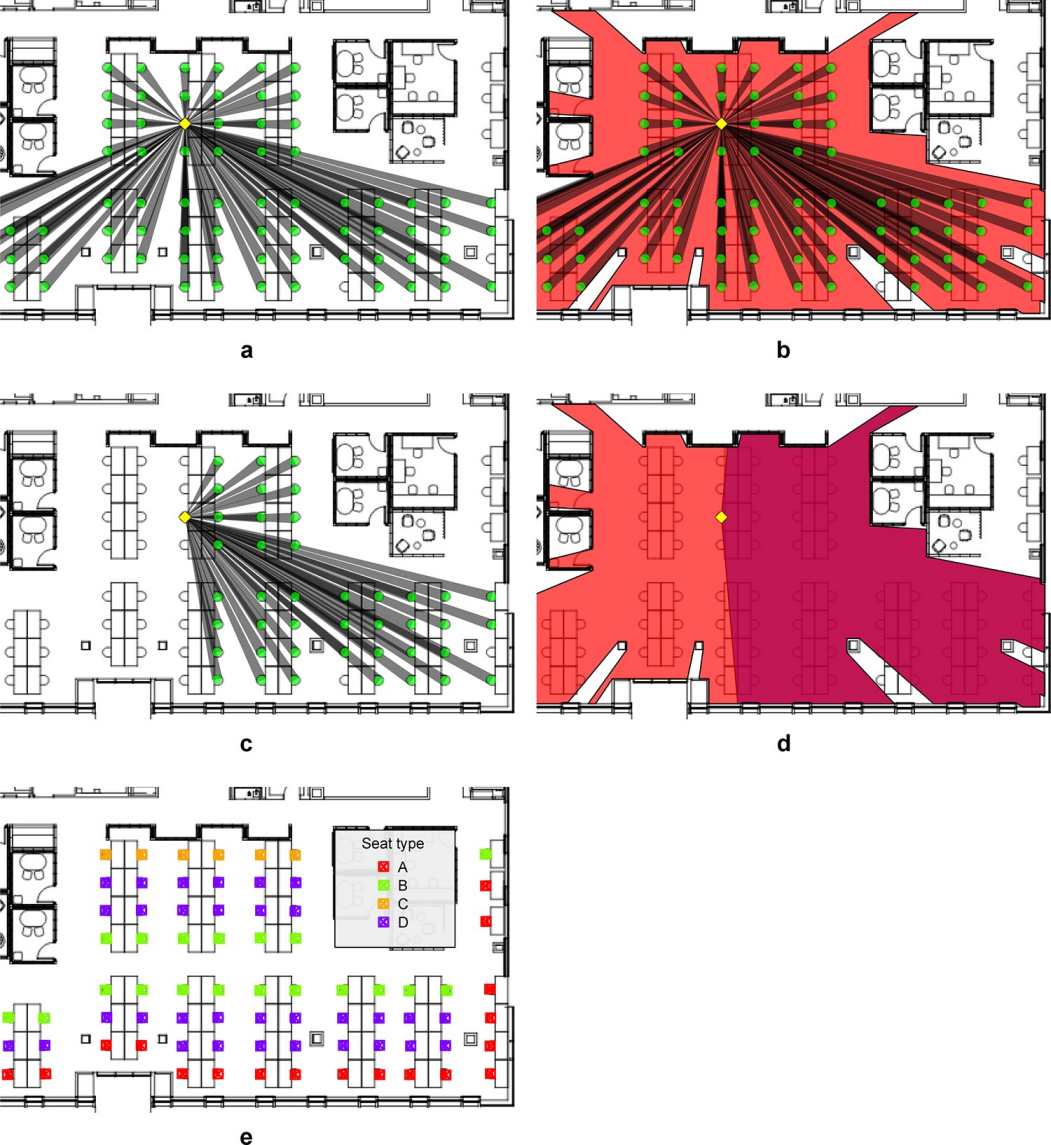
All spatial metrics illustrated for a sample desk and area. a) Degree; b) Density (Degree/Area 360°); c) Outdegree; d) Control (Isovist area 170°/Isovist area 360°); e) Seat types A-D.

Following the explanations on research questions and hypotheses, five detailed spatial metrics were calculated in the analysis. Fig 2 illustrates the metrics for a sample desk and area. The basis for most of the metrics is the seat isovist, a two-dimensional polygon that encompasses the space visible from that seat. The isovist can be either ‘full’ (360°), i.e. including the complete space visible from a seat in a full rotation, or ‘forward facing’ i.e. the space visible if the seat’s direction is taken into account (170°). The metrics used in the analysis were:

- *1) Degree* [n] is a metric borrowed from the field of social network analysis [49]. It denominates the number of people someone is directly connected with. In this context degree shows the number of people potentially sitting in someone’s proximity and are directly visible, i.e. the number of desks within the seat’s 360° isovist (see Fig 2A). Full height partitions or walls were modelled to obstruct visibility, but low furniture was disregarded. Degree therefore defines a metric for co-presence by the number of people someone is potentially surrounded by while sitting at his/her desk.
- *2) Density* [n/m^2^] is a metric of the relative number of others in someone’s desk neighborhood. It is calculated as the ratio of degree and area 360° i.e. the number of desks present in the seat’s full isovist divided by the area of that same isovist (see Fig 2B).
- *3) Outdegree* [n] follows the logic of the homonymous metric in social network analysis which is calculated by counting only the outgoing ties from a person in a directed network. In our case it is calculated as the number of desks directly visible to someone in their 170° forward-facing isovist, constructed from their desk (see Fig 2C). Therefore, it shows the number of people someone can potentially see while facing forward at their desk location.
- *4) Control* [n] is a continuous number between 0 and 1 showing the ratio of the area visible in a 170° forward-facing isovist divided by the area 360° from the same vantage point (see Fig 2D). This is based on extant research, which argued that this metric highlights the degree to which a person feels control over their environment. A value close to 1 denominates that the visible space in their forward-facing isovist (area 170°) is mostly congruent with the visible area around that person (area 360°), i.e. this person is facing the room and can observe any activity happening in the surroundings.
- *5) Seat type* is a categorical metric highlighting the position of the seat in the wider fabric of the desk arrangements (see Fig 2E). As is typical in many contemporary open-plan offices, desks in ‘Tech’ were arranged in double rows of two to four desks length with people opposite facing each other. Four seat types were distinguished in the analysis: A = end of row next to a window (or the atrium); B = end of row next to a main corridor; C = end of row next to a wall; D = mid row.

Initially, nine metrics were defined including for example the area of a full as well as forward-facing isovist in addition to degree and outdegree (counting the number of desks in that same area). Correlation analysis showed high levels of collinearity between some of the metrics, for instance degree was highly correlated with the size of the surrounding area (R^2^ = 0.75) and outdegree was highly correlated with the area of the 170° isovist (R^2^ = 0.85). Therefore, only the five above metrics considered sufficiently distinctive were included in the multiple ordinal regression model. S2 File defines all nine metrics in detail and shows results of the correlation analysis in full.

The next section will present the results of the analysis, firstly showing descriptive statistics for all metrics of interest and then highlighting results from multiple regression modelling.

## Results

### Descriptive statistics and overall insights on working life at ‘Tech’

Table 1 reports descriptive statistics for the four continuous spatial metrics, showing how an average staff member in the open-plan office of ‘Tech’ was surrounded by up to 138 colleagues seated at their desks (mean degree), of which 66 were within someone’s forward-facing view on average (mean outdegree). Variation is large, particularly for outdegree since the person with the highest score could see up to 223 desks in their direct field of visibility. While there were desks that did not see a single other desk in their forward-facing viewshed (minimum outdegree = 0), there was no desk that was not surrounded by at least 17 other desks (minimum degree).

**Table 1:** Descriptive statistics for all continuous spatial metrics including minimum, maximum and mean values as well as standard deviation; sample size n = 167 desks (five survey responses contained missing information on seat type and were excluded from the analysis).

Spatial metric	Unit	Min	Max	Mean	Std Dev
Degree	Number of desks	17	258	137.7	67.7
Density	Number of desks / square meter	0.11	0.28	0.19	0.04
Outdegree	Number of desks	0	223	65.9	50.1
Control	Number	0.01	0.97	0.49	0.26

Overall, densities in the workplace were relatively high with 0.19 desks per square meter, i.e. each desk had a space of around 5 square meters around it. The highest densities were experienced on the fourth floor (see the denser layout in Fig 1), where 0.27 desks per square meter were accommodated, resulting in only 3.7 square meters available per desk in the densest areas.

**Fig 3:**
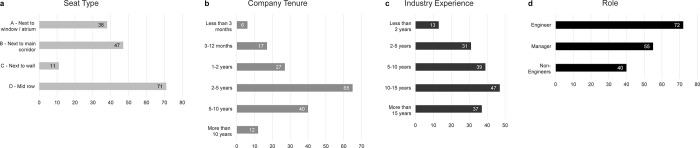
Distribution of categorical variables for study participants. a) Seat Type; b) Company Tenure; c) Industry Experience; d) Role. Roles were aggregated from more detailed role descriptions; non-engineers included roles such as administrators, specialists, data analysts, etc. The final sample size for which we have complete data is n = 167.

Fig 3 gives an overview of distributions of seat type, length of tenure, length of industry experience and role. Seat type D (mid row position) was the most frequent (n = 71), followed by desks next to a main corridor (n = 47) and next to a window (n = 38). Only 7% of desks were positioned next to a wall or a partition (n = 11), which was one of the main features of the workplace design of ‘Tech’. Interior walls were minimized and where they existed, for instance as part of meeting room partitions, desks were normally not placed directly adjacent to them. Length of tenure is roughly normally distributed with the majority of participants having worked at ‘Tech’ for 2–5 years. Experience in the industry is slightly skewed with half of the participants (n = 84) showing more than 10 years of working in technology. Regarding role, engineers (n = 72) form the single biggest group in our sample.

### Multiple ordinal regression models

Multiple ordinal regression models were computed in R to analyze the effects of the independent variables, which included four continuous spatial variables (degree, outdegree, density, control) and one categorical variable (seat type). Tenure and industry experience (ordinal) as well as role (categorical) acted as control variables. Each item in the survey acted as a dependent variable in a separate model, therefore, with ten survey items included in our hypotheses, ten models were computed.

We followed a two-step process, firstly looking at significance and predictors. Five different models showed overall significance, i.e. those predicting satisfaction with sharing information, team identity and cohesion, planned meetings, concentration and working productively. However, degree and density did not produce any significant predictors. This might be explained by the low levels of variation in density and the fact that degree and outdegree still showed some collinearity (r = 0.624) as discussed in S2 File. Therefore, in a second step, we decided to exclude degree and density from the models and computed results again. Outcomes of these reduced models showed the same five survey items as significant. AIC values for the reduced models were slightly lower in comparison and were therefore selected as better fit. Final results are presented in Table 2.

**Table 2:** Output from the multiple ordinal regression models.

Survey items		Accessing coworkers	Sharing information	Awareness of own team	Awareness of other teams	Team identity & cohesion	Spontaneous meetings	Planned meetings	Getting to know people	Concentrating	Working productively
Chi Square		26.162	*33*.026**	25.094	21.417	*34*.858**	10.197	*39*.682**	20.128	*42*.643**	*53*.464**
p-value		0.051	*0*.*007*	0.068	0.163	*0*.*004*	0.856	*0*.*00087*	0.215	*0*.*000*	*0*.*000*
AIC		502.85	*498*.*95*	528.37	551.67	*592*.*05*	631.97	*625*.*77*	560.49	*621*.*84*	*619*.*52*
AIC (null)		497.02	*499*.*98*	521.46	541.09	*594*.*90*	610.16	*633*.*46*	548.62	*632*.*48*	*640*.*98*
**Outdegree**	OR	0.991	0.986**	0.992	1.003	0.989**	0.997	0.987**	1.008	0.982**	0.977**
	p	0.051	**0.002**	0.076	0.522	**0.008**	0.454	**0.004**	0.066	**0.000**	**0.000**
**Control**	OR	13.161**	18.731**	4.072	0.440	11.054**	2.843	5.463	0.369	31.935**	86.055**
	p	0.004	**0.001**	0.110	0.347	**0.005**	0.206	0.052	0.236	**0.000**	**0.000**
**Seat type: B**	OR	0.840	1.286	1.183	0.380*	1.485	1.158	0.848	1.177	1.079	1.178
	p	0.689	0.565	0.700	0.025	0.334	0.725	0.698	0.702	0.857	0.695
**Seat type: C**	OR	0.269	0.260	0.501	1.378	0.369	0.666	1.457	1.929	0.255	0.230*
	p	0.058	0.052	0.297	0.633	0.162	0.541	0.592	0.340	0.054	**0.038**
**Seat type: D**	OR	0.981	1.145	0.848	0.603	0.924	0.869	0.842	1.033	0.953	0.909
	p	0.962	0.736	0.681	0.206	0.837	0.709	0.653	0.933	0.900	0.799
**Tenure: 3–12 months **	OR	0.559	2.022	3.086	0.880	1.557	0.854	2.395	0.471	0.801	0.712
	p	0.514	0.459	0.207	0.886	0.635	0.844	0.301	0.367	0.795	0.699
**Tenure: 1–2 years **	OR	0.239	1.452	5.292	0.215	2.740	0.469	0.697	0.251	0.468	0.410
	p	0.095	0.686	0.051	0.069	0.268	0.329	0.664	0.080	0.349	0.301
**Tenure: 2–5 years **	OR	0.221	0.957	3.563	0.540	1.971	0.549	0.616	0.194*	0.365	0.341
	p	0.057	0.959	0.111	0.439	0.427	0.403	0.525	0.028	0.184	0.180
**Tenure: 5–10 years **	OR	0.179*	0.995	2.936	0.760	1.745	0.515	0.580	0.148*	0.402	0.330
	p	0.039	0.995	0.195	0.741	0.531	0.383	0.492	0.016	0.250	0.185
**Tenure: >10 years **	OR	0.310	1.993	4.815	0.528	1.735	0.539	0.428	0.092**	0.136*	0.141*
	p	0.193	0.482	0.089	0.485	0.571	0.476	0.353	0.007	**0.031**	**0.043**
**Experience: 2–5 years **	OR	0.412	0.561	1.382	1.349	0.902	0.632	2.860	0.629	0.485	0.632
	p	0.198	0.399	0.640	0.675	0.875	0.502	0.125	0.482	0.281	0.490
**Experience: 5–10 years **	OR	0.597	0.446	1.185	2.065	1.212	0.724	1.823	1.540	0.771	0.775
	p	0.449	0.240	0.805	0.289	0.765	0.632	0.362	0.506	0.687	0.692
**Experience: 10–15 years **	OR	0.381	0.373	0.557	1.833	0.647	0.775	2.508	1.140	0.831	0.960
	p	0.153	0.149	0.393	0.378	0.500	0.710	0.171	0.837	0.782	0.951
**Experience: >15 years **	OR	0.276	0.222*	0.625	1.551	0.635	0.728	1.705	0.898	0.655	0.743
	p	0.055	**0.028**	0.492	0.519	0.484	0.639	0.427	0.867	0.531	0.661
**Manager **	OR	0.918	1.630	1.969	1.162	2.055*	1.588	1.824	1.888	2.265*	2.450*
	p	0.808	0.174	0.054	0.674	**0.035**	0.187	0.084	0.068	**0.021**	**0.011**
**Non-Engineer**	OR	1.147	1.803	2.296*	2.014	3.771**	1.836	2.353*	2.023	2.280*	2.041
	p	0.727	0.137	0.037	0.075	**0.001**	0.107	**0.028**	0.071	**0.036**	0.065

The Akaike Information Criterion AIC is reported as a goodness of fit statistic alongside the Model Chi Square. Odds ratios (OR) and p-values are reported for all variables. Seat type A, shortest tenure and experience as well as the role of an engineer were selected as baseline. Insignificant predictors and models are shown in grey. Significant predictors for significant models are shown in bold. Significance levels of p<0.05 are highlighted by * and p<0.01 by**.

Satisfaction with sharing information, team identity and cohesion, planned meetings, concentrating and working productively were highly significantly predicted by the variables. Model Chi Square values are highest for concentrating and working productively, which means the variables included in the models make the most difference to these aspects of staff satisfaction ratings.

The three control variables show some significant effects. Compared to staff in their first three months working at ‘Tech’, those with a tenure longer than ten years are approximately seven times less likely to give favorable ratings for concentrating (0.136) and working productively (0.141). Compared to newcomers to the industry, people with more than 15 years of experience are 4.5 times less likely to rate their satisfaction with sharing information high (0.222). Finally, compared to an engineer, those in the role of managers and non-engineers are 2–4 times more likely to rate their environment positively regarding team identity and cohesion (2.055 and 3.771), planned meetings (non-engineer: 2.353), concentrating (2.265 and 2.280) and working productively (manager: 2.450).

Considering seat types, workers seated next to a wall are significantly less satisfied with their ability to work productively than those seated next to a window with odds for a positive rating reduced by a factor 4.3 (OR = 0.230).

In order to make sense of the size effect of the continuous variables outdegree and control, as a baseline figure half a standard deviation of each spatial metric was used as an exemplary change to someone’s workplace environment. Effects are also reported for the comparison between an average workplace and the most extreme one found in the study. Results are summarized in Table 3.

**Table 3:** Effects from the odds ratio for all significant continuous variables from the ordinal regression models.

Spatial metrics				Survey items	OR	Difference in survey response	
Item	Mean	Std Dev	Max			With ½ Std Dev	Max–Mean
Outdegree	65.9	50.1	223	Sharing information	0.986	-35%	-220%
				Team identity and cohesion	0.989	-28%	-173%
				Planned meetings	0.987	-33%	-204%
				Concentrating	0.982	-45%	-283%
				Working productively	0.977	-58%	-361%
Control	0.49	0.26	0.97	Sharing information	18.731	231%	851%
				Team identity and cohesion	11.054	131%	483%
				Concentrating	31.935	402%	1485%
				Working productively	86.055	1106%	4083%

Differences in survey responses are calculated for half a standard deviation on the spatial metric as well as the difference between mean and maximum values.

Outdegree showed a mean value of 66, so an average person seated at their desk had 66 other desks within their visual field. If that value was increased by half a standard variation, the odds of rating the workplace supportive of sharing information would drop by 35%. Likewise, the odds for satisfaction with team identity and cohesion would decrease by 28%, and by 33% for planned meetings. Concentration and working productively are most affected since the odds for a favorable rating would decrease by 45% and 58% respectively.

The maximum outdegree value observed is large with 223 desks included in someone’s visual field; therefore, the difference in survey responses between average and maximum values are considerable. The person seated at the desk with maximum outdegree is 220% less likely to rate the environment favorable for sharing information, 173% less likely regarding team identity and cohesion, 204% for planned meetings, 283% for concentrating and even 361% for working productively.

Control has a mean value of 0.49, almost exactly at half point, which is due to the regular workplace layout of ‘Tech’. If the control ratio was increased by half a standard variation, the odds of rating the workplace supportive of sharing information would rise by 231%. Likewise, the odds for satisfaction with team identity and cohesion would increase by 131%. Concentration and working productively are again most significantly affected, since the odds for a favorable rating would increase by 402% and 1105% respectively.

The maximum control value observed is 0.97, which means this person visually controls almost the whole environment they are surrounded by, or in other words, their forward-facing and surrounding areas are almost the same. A person with maximum control is 851% more likely to rate the environment favorable for sharing information, 482% more likely regarding team identity and cohesion, 1485% for concentrating and even 4083% for working productively. In other words, with highest visual control, workers are 40 times more likely to rate productive working favorably.

## Discussion

Bringing results together and comparing them to the hypotheses introduced earlier, we will go into individual findings as supported by the regression models, but also conceptualize the survey items into four broader aspects of work: 1) teamwork (accessing coworkers, sharing information, team identity and cohesion, spontaneous and planned meetings, getting to know people); 2) awareness (of own and other teams); 3) focused work (concentrating) and 4) perceived productivity at work. Table 4 provides an overview of the hypotheses and whether they are accepted or rejected by our results.

**Table 4:** Summary of supported and rejected hypotheses.

Hypothesis	Spatial quality	Effect	Result
H1a	Higher degree	Positive rating on teamwork	Rejected
H1b	Higher density	Positive rating on teamwork	Rejected
H2	Higher degree	Positive rating on awareness	Rejected
H3a	Lower degree	Positive rating on team identity, focused work and perceived productivity	Rejected
H3b	Lower outdegree	Positive rating on team identity, focused work and perceived productivity	Accepted
H4a	Higher control	Positive rating on teamwork	Accepted
H4b	Higher outdegree	Positive rating on teamwork	Rejected
H5a	Seat type next to corridor	Positive rating on teamwork	Rejected
H5b	Seat type next to window	Positive rating on focused work and perceived productivity	Partially accepted

Hypotheses H1a, H1b and H5a suggesting positive ratings on teamwork with higher degree, higher density and a seat next to the corridor were rejected due to not reaching significance. This means we could not find support for the findings of Allen [25] regarding the ease of reaching coworkers via density or higher numbers of people in someone’s proximate surroundings; neither for the findings of Backhouse and Drew [16] regarding the benefits of passing traffic next to desks. Likewise, H2 was rejected due the relevant models being insignificant, meaning we could not find support for the work of Henn and Allen [35] on awareness. H3a is rejected for the same reason (no significance), which means the effects described by scholars such as Kim and De Dear [14] or Danielsson and Bodin [24] on privacy are not related to the overall size of the surrounding neighborhood. Instead, we found support for hypothesis H3b on the size of someone’s forward-facing viewshed as defined by visibility graphs and isovist analysis [50, 51], as those with lower outdegree were more likely to rate team identity and cohesion, concentration and productive work higher. Interestingly, while our results are in line with scholars interested in visual privacy [14], it contradicts recent findings by Alavi et al. [52], who found that seats with large forward-facing viewsheds were preferred in a highly visually connected open-plan office, possibly due to the fact that their case study was rather small (33 participants).

Further, hypothesis H4b is rejected because it showed the opposite effect to what we assumed and what the literature might suggest. A higher outdegree does not lead to positive ratings of teamwork; instead we find that smaller viewsheds supported perceptions of teamwork (sharing information, team identity and cohesion, planned meetings). Our data suggests similar positive effects for teamwork (sharing information, team identity and cohesion) for those workers with higher levels of control. Therefore, H4a is confirmed in line with the literature on the preferential choice of seats in café style environments [36].

H5b is only partially supported, as we hypothesized that a seat next to a window would result in higher ratings for focused work. This is only the case if seats next to a window are compared to those next to a wall (which reduces the odds of considering the working environment productive), but is not the case in comparison to seats next to a corridor or mid row. These results could be interpreted in favor of window seats, partially underlining the effects found by Kim and De Dear [38] or could possibly manifest a disadvantage that seats next to walls might bring.

In summary, while awareness for others did not yield results in our analysis, we can confirm that indeed office workers at ‘Tech’ perceived teamwork, focused work and productivity differently according to the spatial characteristics of their desks while controlling for other intervening variables such as role, tenure and industry experience. We found two spatial factors particularly relevant: how many other desks someone permanently has in their line of sight, and how much someone’s back is protected.

Having fewer people in sight and feeling more in control of the environment by facing the room resulted in significantly higher odds for positive ratings of focused work and perceived productivity. Generally speaking, effect sizes of control were larger throughout than of outdegree. The effects on focused work are in line with earlier studies which alluded to the fact that larger open-plan areas compromised satisfaction and concentrated work [11–13, 24]. Our findings articulate these insights and add nuance to our understanding in this area. It was not only the size of an area that staff found troublesome, but more so the effect of seeing many others (hence being exposed to potential distractions) in conjunction with low levels of control (hence being excluded by activities behind someone’s back). These findings are particularly relevant for the emerging focus on accommodating neurodiversity in the workplace [53].

More surprisingly perhaps with fewer desks in someone’s forward-facing viewshed, workers were more likely to also rate teamwork highly. Potentially seeing a large number of people and facing away from the room showed negative effects on sharing information with others, but also on one’s own team identity. Sharing information might have been inhibited by the strong sense of not disturbing others close by (in the case of high outdegree), or simply not seeing who is around (in the case of low control). Team cohesion might have suffered due to a lack of boundaries between teams in larger areas and a feeling that spaces were not tailored to specific needs. Those facing away from rooms might have members of their team not directly visible. The fact that planned meetings were perceived negatively when seeing more desks is slightly more puzzling, since the provision of meeting rooms was uniform across the office space. We can only speculate why this would be the case. One possible explanation might be that being able to see more colleagues directly took out some necessity for planned meetings.

Open-ended feedback collected via the questionnaire underlines some of the above-mentioned points. Staff at ‘Tech’ show good intuition into how their workspace operates and the effects it might have on their work: *“Open space in my area is far too large/noisy*. *I can literally see/hear 100+ people from my desk”*, or: *“Because the office is so open it feels hard to talk to colleagues in the open-plan area without disturbing everyone”*. They even realize that visual distractions are crucial: *“While noise is not a great issue (it is an issue though)*, *visual distractions are pretty bad*. *I would much prefer cubicles or some other forms of walls to split open space into smaller areas with no visual contact*. *Seeing all my teammates and other teams does not really help me cooperate with them as I either block out sound with headphones or try to ignore it”*. Another worker makes it equally explicit: *“Can we have offices with walls and doors*, *please*? *The open-plan layout is extremely noisy and distracting*. *I work primarily with my immediate team*, *most people do*. *A range of office sizes from 4 to 10 people would allow many teams to sit together without distraction from other people they don’t actually work with*. *This would improve productivity*, *allow people to stop wearing headphones all day long*, *and form a stronger team identity because they have a more clearly defined space*.*”*

In the case presented here of a globally operating technology company, having many colleagues in direct sight and plenty of activities going on behind someone’s back, as is typical for large open-plan offices was not found as favorable as being in smaller, more easily controllable areas. This finding somewhat contradicts the research of Allen [25] as argued above but also later research in the same vein [54, 55], which made a strong case for proximity as a factor facilitating exchange and teamwork by showing that communication and collaboration fell with distance, as well as with changes in floors or buildings. In order to minimize distance, tightly packed and large open-plan offices became the preferred mode of operation for many organizations. We do not dispute the importance of proximity per se, yet it must be concluded that bigger space is not automatically better. In our case it resulted in higher odds of rating the environment negatively.

### Implications for workplace science and design

These findings have wide ranging implications for both theory and practice of workplace science and workplace design.

From a theoretical point of view, this study showed the importance of human vision and the effects different properties of the visual field can have on one’s satisfaction with the workplace. This study therefore builds significantly on the work of others who maintained that configuration matters for satisfaction ratings in the workplace [33], yet to our knowledge this is the first such study using visibility graphs and visual fields successfully.

Implications for the practice of workplace design are also relevant. Over the last years, many large technology companies have planned or built ‘cathedrals’ of interaction [56], mainly in the Silicon Valley, such as the new Apple HQ in Cupertino (designed by Foster and Partners), the Samsung America HQ in San Jose (designed by NBBJ), the Facebook HQ in Menlo Park (designed by Frank Gehry), or the planned Google HQ in Mountainview (designed by BIG and Heatherwick) [57]. All of those designs boast large open floor plates in an attempt to support encounters and collaboration essential to knowledge work. Our results, however, suggest that a more nuanced approach to openness might be preferable, as increased forward-facing views induced by large open-plan office design have negative effects, as they are impacting staff satisfaction of teamwork, focused work and perceived productivity. Therefore, designing smaller and more intimate areas might be advisable as an immediate workplace design choice. If large areas are unavoidable, giving workers control by allowing them to face the room as much as possible can mediate negative effects on teamwork and focused work. Even with the role of physical offices under scrutiny due to the long-lasting effects of Covid-19 and an increased appetite for working from home, these insights deliver relevant principles for designing workplaces in a way that they attract staff back to the office.

## Conclusions

This paper has reported results from a single case study of the workplace of a technology company in London. It was found that the micro-location of a desk within the wider fabric of the office layout has effects on how likely it is that the person occupying this desk will rate their environment favorably regarding different aspects of teamwork, focused work and perceived productivity while controlling for other factors potentially impacting satisfaction. As such this study stands in the tradition of environmental psychology studies on satisfaction at work, but due to working with visibility related metrics it also contributes to space syntax research.

In detail, higher outdegree, i.e. a higher number of desks in someone’s forward facing view made it less likely for the environment to be considered supportive of team identity, sharing information, meeting others in planned ways, concentrating on tasks and working productively. The same effects (yet not for planned meetings) were evident for lower levels of control, i.e. seeing fewer desks in relation to the number of desks someone is surrounded by. A seat next to the wall was perceived as less favorable for productive work than a seat next to the window.

As every piece of research, this paper has limitations, mainly the fact that insights were only derived from a single case. Only repeat studies will be able to tell whether these insights are generalizable. Including other companies from the technology industry, but also across different industries would be worthwhile to add nuances to our understanding of the impact of visibility effects in the office. Another limitation is the relative homogeneity of floor plans at ‘Tech’. Cases with more variance in spatial metrics might yield additional results. Particularly the effects of density were hard to pin down due to the relative uniformity of office accommodation in the study at hand. Further research might also include gender as a control variable. Finally, more advanced multivariate models also considering interaction effects between variables could provide a more accurate understanding of how built environment factors interact with behavioral choices and preferences, following models proposed in extant research [58].

To conclude, this study has contributed to a nuanced understanding of the effects of visibility on workplace satisfaction and the way in which the environment supports different work activities, ranging from teamwork to focused work. Satisfaction with the workplace can be concluded to be differential, since it depends on the characteristics of a desk and the spatial qualities associated with visible fields.

## Supporting information

S1 File (DOCX)

S2 File (DOCX)
