# Supplementary material for: Differential perceptions of teamwork, focused work and perceived productivity as an effect of desk characteristics within a workplace layout
Source: PLoS One. 2021 Apr 28;16(4):e0250058. doi: 10.1371/journal.pone.0250058 (PMC8081188; doi:10.1371/journal.pone.0250058)
Supplement: S1 File — (DOCX) [file pone.0250058.s001.docx]

**Your Workspace**

1. Which is your desk? Please find the sticker on your desk showing your desk ID number (5 digits with letters and numbers) and type it into the box below. This will allow us to analyse your location in the office, for instance your viewshed in relation to your answers in the survey. We don’t have a list of names against desks, so won’t be able to identify anyone from this information.

**The Role of the Workspace: Supportive or Inhibitive?**

1. How much does the workspace at XXXXXXX support or inhibit the below listed aspects of working life? Please rate the role of the workspace for each on a scale from +3 (extremely supportive) to -3 (extremely inhibitive).

|  | Extremely supportive | Very supportive | Fairly Supportive | Neutral | Fairly Inhibitive | Very Inhibitive | Extremely Inhibitive |
| --- | --- | --- | --- | --- | --- | --- | --- |
|  | **+3** | **+2** | **+1** | **0** | **-1** | **-2** | **-3** |
| Accessing people you work with regularly |  |  |  |  |  |  |  |
| Sharing information with others quickly |  |  |  |  |  |  |  |
| Knowing what is going on in your team |  |  |  |  |  |  |  |
| Knowing what is going on in other teams |  |  |  |  |  |  |  |
| Your team’s identity and cohesion |  |  |  |  |  |  |  |
| Meeting others spontaneously |  |  |  |  |  |  |  |
| Meeting others in planned ways |  |  |  |  |  |  |  |
| Getting to know people in the office |  |  |  |  |  |  |  |
| Visiting other floors |  |  |  |  |  |  |  |
| Concentrating on tasks |  |  |  |  |  |  |  |
| Getting your job done productively |  |  |  |  |  |  |  |

**About You**

1. How long have you worked at XXXXXXX?

Less than three months

3 months to 12 months

1-2 years

2-5 years

5-10 years

More than 10 years

1. How long have you worked in the industry?

Less than 2 years

2-5 years

5-10 years

10-15 years

More than 15 years

1. What is your role?

Non-Manager Software Engineer

Manager

Product Manager

Technical Program Manager

User Experience

Other

1. Do you have any other comments for us on the study, the role of the workspace, your office environment and your happiness at work?

Thanks a lot for your time in answering the questions.

If you want to be kept informed of the findings of the study, please email XXXXXXXX with the subject line: XXXXXXX Flexible Spaces – Findings and tell us you’re interested.
