# Supplementary material for: Differential perceptions of teamwork, focused work and perceived productivity as an effect of desk characteristics within a workplace layout
Source: PLoS One. 2021 Apr 28;16(4):e0250058. doi: 10.1371/journal.pone.0250058 (PMC8081188; doi:10.1371/journal.pone.0250058)
Supplement: S2 File — (DOCX) [file pone.0250058.s002.docx]

**S2 File FOR PLOSONE PAPER**

*Differential perceptions of teamwork, focused work and perceived productivity as an effect of desk characteristics within a workplace layout – Kerstin Sailer, Petros Koutsolampros, Rosica Pachilova*

SPATIAL METRICS

The study initially defined nine spatial metrics potentially of interest. Figure S1 shows a visual explanation for all metrics based on a sample desk.


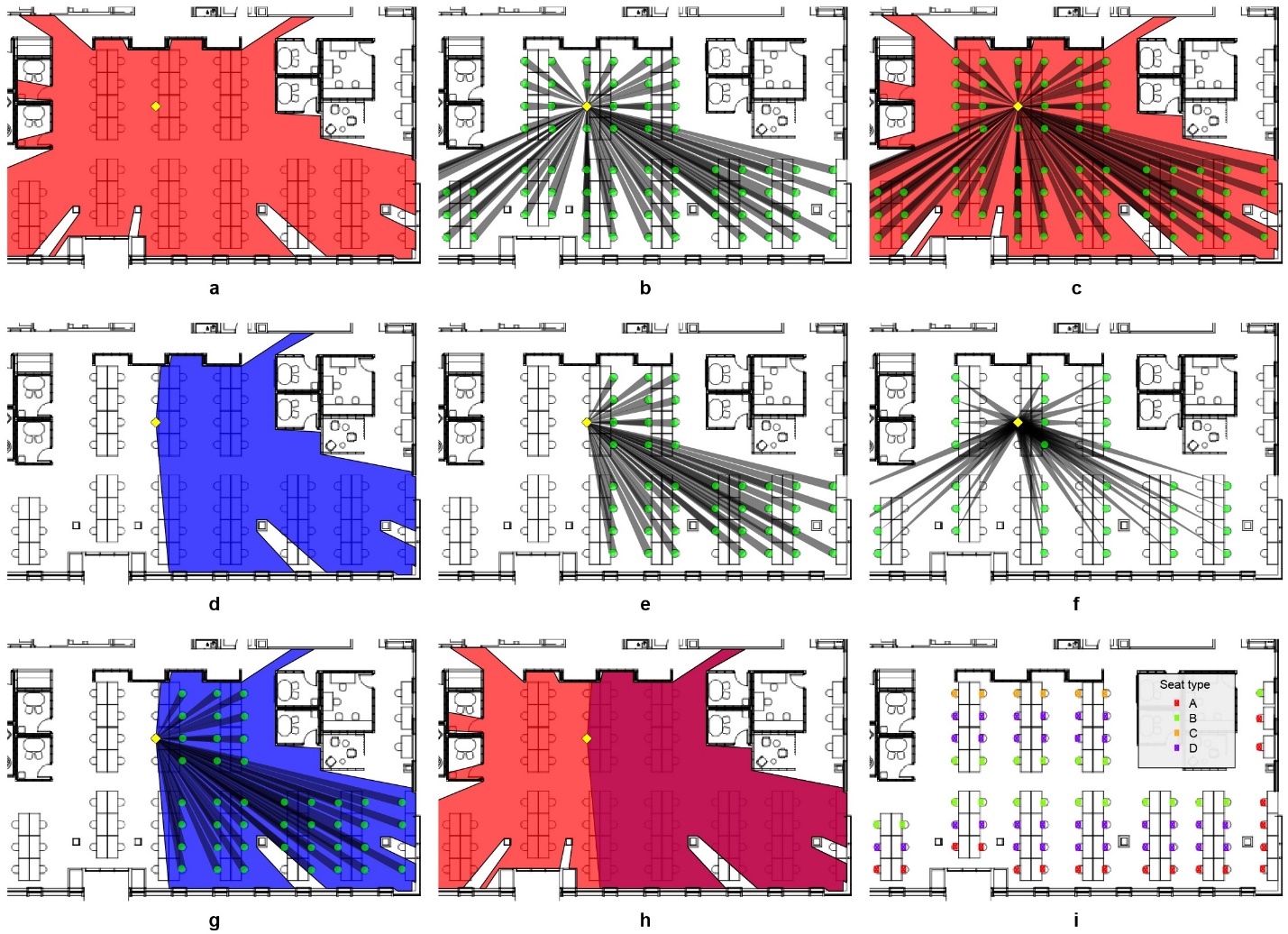


**Fig S1.** All spatial metrics illustrated for a sample desk and area. a) Isovist area 360°; b) Degree; c) Density (Degree/Area 360°); d) Isovist area 170°; e) Outdegree; f) Indegree: g) Forward-facing density (Outdegree/Area 170°); h) Control (Isovist area 170°/Isovist area 360°); i) Seat types A-D.

Only five metrics were used in the main paper. This supplementary material introduces and defines all nine metrics in detail and shows in depth why only five were used in the analysis.

The following spatial metrics were initially calculated and analysed:

- **Area 360° [m2]** is a proxy metric for the size of a neighborhood in the office (see figure S1a), calculated as the area of the full isovist of each seat. Full height partitions or walls were modelled to obstruct visibility, but low furniture was disregarded. With desks sometimes arranged around corners it was not straightforward to determine where one neighborhood started and ended. Thus, this proxy was used, which resulted in a slightly different neighborhood size for each desk, but desks next to each other with similar visibility properties would have similar neighborhood sizes. The area of the floor plate was disregarded as a metric since it showed almost no variation across the floors.
- **Degree [n]** is a metric borrowed from the field of social network analysis. It denominates the number of people someone is directly connected with. In this context degree shows the number of people potentially sitting in someone’s proximity and are directly visible, i.e. the number of desks within the seat’s 360° isovist (see figure S1b). It therefore defines a metric for co-presence by the number of people someone is potentially surrounded by while sitting at his/her desk.
- **Density [n/m2]** is a metric of the relative number of others in someone’s desk neighborhood. It is calculated as the ratio of degree and area 360° i.e. the number of desks present in the seat’s full isovist divided by the area of that same isovist (see figure S1c).
- **Area 170° [m2]** is a metric similar to the area around someone’s desk, but rather than considering a full isovist, it is calculated by only taking the forward-facing (targeted) isovist into account (see figure S1d). The metric essentially maps the visible area while seated, taking into account the desk orientation but also once again only modelling high partitions as obstacles and ignoring low level furniture.
- **Outdegree [n]** follows the logic of the homonymous metric in social network analysis which is calculated by counting only the outgoing ties from a person in a directed network. In our case it is calculated as the number of desks directly visible to someone in their 170° forward-facing isovist, constructed from their desk (see figure S1e). Therefore, it shows the number of people someone can potentially see while facing forward at their desk location.
- **Indegree [n]** counts the numbers of incoming ties in a directed network. In this context indegree denominates the numbers of people who can see the desk of a person while they are sitting at their own desks, forward-facing. Thus, it shows ‘being seen’ rather than seeing, or in other words, the potential number of people that face someone (see figure 1f).
- **Density – forward-facing [n/m2]** is a metric of the relative number of people in someone’s forward-facing field of visibility (see figure S1g). It is calculated as the number of seats present in a 170° isovist (outdegree) divided by the area of that same isovist (area 170°).
- **Control [n]** is a continuous number between 0 and 1 showing the ratio of the area visible in a 170° forward-facing isovist divided by the area 360° from the same vantage point (see figure S1h). This is based on extant research, which argued that this metric highlights the degree to which a person feels control over their environment. A value close to 1 denominates that the visible space in their forward-facing isovist (area 170°) is mostly congruent with the visible area around that person (area 360°), i.e. this person has a protected back and can observe any activity happening in the surroundings.
- **Seat type** is a categorical metric highlighting the position of the seat in the wider fabric of the desk arrangements (see figure S1i). As is typical in many contemporary open-plan offices, desks in ‘Tech’ were arranged in double rows of two to four desks length with people opposite facing each other. Four seat types were distinguished in the analysis: A = end of row next to a window (or the atrium); B = end of row next to a main corridor; C = end of row next to a wall; D=mid row.

In order to use the spatial metrics in a multiple ordinal regression as predictors, we needed to select those that offer distinct information. It is easy to see how for example the size of a surrounding desk neighborhood is depicted both by isovist area (figure S1a), but also by degree (figure S1b), i.e. the numbers of desks counted in that area. Likewise, forward-facing isovist area (figure S1d) and outdegree (figure S1e), i.e. numbers of desks within forward-facing viewshed match up to a high degree. Collinearity is an issue here, as the correlations of all continuous metrics highlight. Variable distributions and correlation scattergrams as well as the r correlation coefficients are presented in figure S2 below.


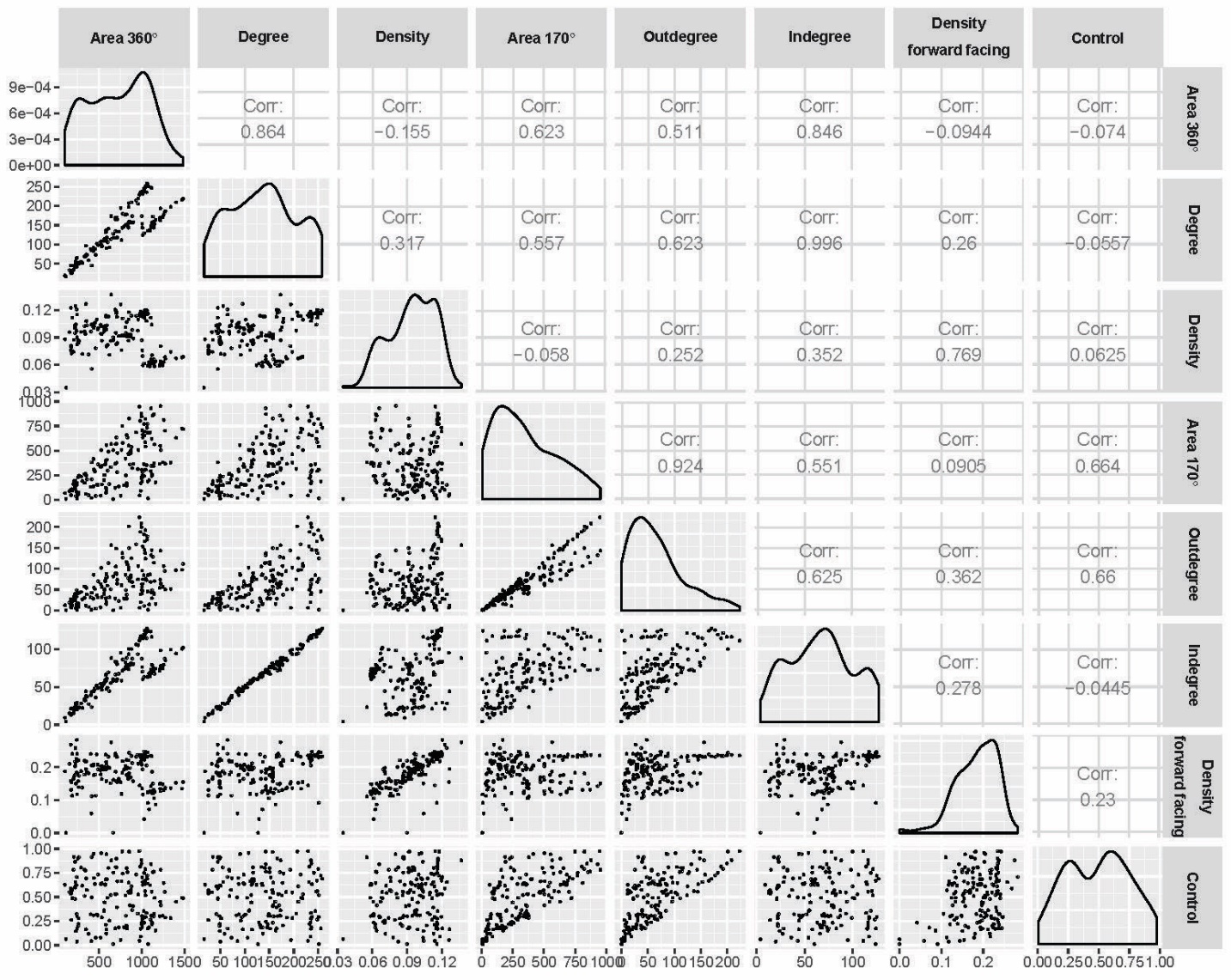


**Fig S2. Overview of all continuous spatial variables** based on all desks for which survey responses exist (n=167). The diagram shows distribution curves, as well as scattergrams and Pearson correlation coefficients r for how variables interrelate.

As already alluded to, area of an isovist and numbers of desks in that same area are highly correlated, as expected: area 360° and degree correlate with r=0.864, thus R^2^=0.75. Area 170° and outdegree correlate with an even higher coefficient of r=0.924, thus R^2^=0.85. Therefore, we have decided to only keep degree and outdegree as relevant variables for the multiple regression modelling, as they more closely align with our hypotheses – we believe it is the numbers of people present that makes a difference to people’s teamwork and focused work perceptions, for which the numbers of desks is a better proxy than the visible area. So, area 360° and area 170° were discarded. Keeping degree as a variable also meant discarding indegree, as those two metrics show the highest level of collinearity with r=0.996 and R^2^=0.99. The reason for this almost perfect correlation lies in the regular desk layout. The numbers of desks someone is surrounded by when sitting at their desk is roughly twice the size of the numbers of people one is seen by (forward-facing), which is due to rows of desks arranged in the same orientation and half of the workers facing one way, with the other half facing the other way. We preferred degree as a metric showing the overall numbers of desks, giving a proxy for the amount of co-present people to the metric indegree (as a proxy for being seen), hence indegree was left out of the further modelling. Density and forward-facing density also correlate as expected with r=0.769 and R^2^=0.59. Density has the more normal distribution, hence was preferred to forward-facing density. Seat type is derived from micro-locations and is therefore by nature independent of the isovist-derived metrics.

This leaves us with five spatial metrics included in the overall modelling of the main paper: 1) degree, 2) density, 3) outdegree, 4) control, 5) seat type. The former four show the following correlation coefficients R^2^ among each other (in decreasing order): R^2^=0.44 (outdegree and control), R^2^=0.39 (degree and outdegree), R^2^=0.10 (degree and density), R^2^=0.06 (density and outdegree), R^2^=0.004 (density and control), R^2^=-0.003 (degree and control). The two highest correlations of this set (outdegree versus control and degree versus outdegree) only show this level of collinearity because the lower half and upper half of the respective scattergrams are empty. This means there are some seats, especially at the edges of the floor plan for desks facing the room, where the values for degree and outdegree are almost the same, or likewise, outdegree is high and control is high. However, towards the centre of the floor plate, seats will show higher variation between the variables.

Therefore, these five spatial metrics were considered distinctive enough in the information they contribute for simultaneous inclusion in the multiple regression modelling.
